# Mucin-1 is required for Coxsackie Virus B3-induced inflammation in pancreatitis

**DOI:** 10.1038/s41598-019-46933-y

**Published:** 2019-07-23

**Authors:** Xiang Liu, Dahn L. Clemens, James A. Grunkemeyer, Jeffrey D. Price, Kelly O’Connell, Nora M. Chapman, Peter Storz, Haitao Wen, Jesse L. Cox, Whitney L. Reid, Michael A. Hollingsworth, Sarah Thayer

**Affiliations:** 1 Division of Surgical Oncology, Department of Surgery, Fred and Pamela Buffet Cancer Center, University of Nebraska Medical Center, Omaha, NE USA; 2 Department of Internal Medicine, Division of Gastroenterology and Hepatology, University of Nebraska Medical Center, Omaha, NE USA; 3 Midwest laboratories, Omaha, NE, University of Nebraska Medical Center, Omaha, NE USA; 4 Eppley Institute for Research in Cancer and Allied Diseases, Fred & Pamela Buffett Cancer Center, University of Nebraska Medical Center, Omaha, NE USA; 5 Department of Pathology and Microbiology, University of Nebraska Medical Center, Omaha, NE USA; 6 Department of Cancer Biology, Mayo Clinic, Jacksonville, FL 32224 USA; 7 Department of Microbial Infection and Immunity, Infectious Disease Institute, The Ohio State University Comprehensive Cancer Center, The Ohio State University, Columbus, OH 43210 USA

**Keywords:** Gastroenterology, Pancreatic disease

## Abstract

The Muc-1 oncoprotein is a tumor-associated mucin often overexpressed in pancreatic cancer. We report that knockout of Muc-1 reduced the degree of pancreatic inflammation that resulted from infection with Coxsackievirus B3 (CVB3) in a mouse model. CVB3-infected Muc-1-deficient (Muc-1^KO^) mice had significantly reduced infiltration of macrophages into the murine pancreas. We found that Muc-1 signaling through NF-κB increased expression of ICAM-1, a pro-inflammatory mediator that recruits macrophages. Further investigation revealed that bone marrow derived macrophages (BMDM) from the Muc-1^KO^ mice exhibited defective migration properties, in part due to low expression of the C-C motif chemokine receptor (CCR2) and the integrin Very Late Antigen 4 (VLA-4). The results presented here provide novel insight into the role of Muc-1 in regulating the inflammatory response and the cellular microenvironment in pancreatitis.

## Introduction

Mucins are heavily glycosylated proteins that give the mucosa viscous gel-forming properties, and contribute to the structural organization of secretory epithelia. The mucosa serves as a physical barrier to protect the epithelium from harsh environments such as low pH and microbial infections. Cell surface associated mucins, such as Mucin-1 (Muc-1), are important components of the mucosal barrier that exist at the interface of the apical surface between secreted mucins and the cell surface^1–4^. In addition to the mucus-type functions of its extracellular mucin domain, Muc-1 is a transmembrane protein with a cytoplasmic tail that is a known target of multiple kinases that has been shown to participate in normal and oncogenic signaling^1–4^. Muc-1 is overexpressed and aberrantly glycosylated in many cancers^1–4^, where it has been documented to play an important role in inflammation and tumor progression^1–4^. Muc-1 is also expressed on many non-epithelial cell types including leukocytes, where its function is less well characterized. It has been documented that Muc-1 controls inflammation resulting from Helicobacter pylori infection by suppressing activation of the NLRP3 inflammasome^5,6^. Knockout of Muc-1 also results in aberrant expansion of myeloid derived suppressor cells from the bone marrow^7^.

Group B coxsackieviruses (CVB), common and medically important human pathogens, are a frequent cause of acute and chronic inflammatory disease of human pancreas^8,9^. Infections with coxsackievirus can cause acute inflammatory pancreatitis in mice that is similar to human disease^8,10–14^. The contribution of coxsackievirus B3 to the progression of acute pancreatitis has been documented in mice^14,15^. We elected to use this unique and physiologically relevant model of pancreatitis (infection with CVB3) to study the progression of early stages of pancreatitis, specifically to study the role of Muc-1 in this inflammation model.

Here, we explored potential functions of Muc-1 in inflammation that accompanies CVB3-induced pancreatitis, and discovered that mice lacking Muc-1 showed a dramatically attenuated inflammatory and tissue remodeling reaction to virus infection. Further investigation revealed that inflammatory macrophages from Muc-1-deficient mice exhibited defective migration properties, in part due to low expression of the chemokine receptor CCR2 and the integrin Very Late Antigen 4 (VLA-4). These results provide insight into the role of Muc1 and its regulation of key inflammatory cells in virally-induced pancreatitis. These findings suggest that Muc1 is a novel molecular target for intervening in cases of acute inflammatory pancreatitis.

## Results

### Muc-1 accentuates coxsackie virus B3-induced pancreatitis

**Figure 1:**
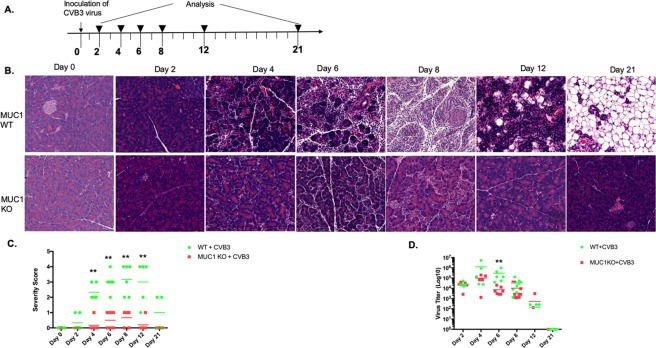
Coxsackie virus B3 infected Mu-c1^KO^ mice fail to develop severe pancreatitis. (**A**) Treatment scheme for CVB3. Wildtype (WT) control and Muc-1^KO^ mice were infected with CVB3 (n = 8 uninfected and n = 55 infected for each mouse strain). Each experiment was repeated four times. Half of the mice from each group were randomly selected for evaluation of virus titer and severity score. (**B**) Representative H&E-stained sections of pancreata from wildtype mice (upper panels) and Muc-1^KO^ mice (lower panels) inoculated with CVB3 for 0, 2, 4, 6, 8, 12, 21 days. (**C**) Virus titer from spleens following virus infection at each time point was determined by plaque assay. (**D**) Severity score was graded on acinar cell loss, edema, and inflammation (leukocyte infiltration). A score of 1 = 5–25%, 2 = 26–50%, 3 = 51–75%, 4 = 76–100% of the total pancreas. Each data point indicates the severity score of an individual mouse. Severity score was graded by two different blinded pathologist residents (JC and WR).

To evaluate the role of Muc-1 and its signaling in the progression of a physiologically relevant model of acute inflammatory pancreatitis, WT and Muc-1^KO^ mice were infected with CVB3 (Fig. 1A). Similar to previous studies^13,14,16,17^, we found that WT mice infected with CVB3 display severe inflammatory pancreatitis (characterized by inflammatory cell infiltration and acinar cell necrosis) that was evident by day 4 post-infection, peaked at days 6–8, and was resolved with evidence of some fatty replacement by day 21 (Fig. 1B upper panels). In striking contrast, Muc-1^KO^ mice developed only mild inflammatory responses and greatly attenuated pancreatitis during the 21-day time course (Fig. 1B bottom panels). We repeated these experiments inoculating mice with higher titers of virus; results showed that even when viral titers were extremely high at the peak of infection in both strains (10^6^ to 10^7^ pfu), the same trend in histology was observed (little inflammation and pancreatitis in the Muc-1^KO^ mice) in 4 independent experiments (Supplementary Fig. 1A,B). An inflammation severity score, based on the percentage of pancreas with acinar cell loss, edema, and infiltrating immune cells showed significantly less inflammation in CVB3 infected Muc-1^KO^ mice as compared to WT infected mice (Fig. 1C). The body weight of the mice with or without infection remained at similar levels at all time points (Supplementary Fig. 1C). However, the wildtype mice had significant pancreatic weight loss at 8 days post-infection compared to the wildtype non-infected mice (Supplementary Fig. 1D). We confirmed Muc-1 expression during pancreatic injury by immunofluorescence on tissue collected from CVB3 infected WT and Muc-1^KO^ mice pancreata 8-days post-infection (Supplementary Fig. 1E). Muc-1 is normally detected in acinar cells and all ducts including intercalated ducts, intra-lobular ducts and interlobular ducts^18^ (Supplementary Fig. 1F). At 8-days post-infection in Muc-1 wildtype mice, when most acinar cells were lost, Muc-1 expression was detected in remaining ductal structures. In addition to its expression in normal epithelial cells, Muc-1 is also expressed in macrophages (Supplementary Fig. 1G).

To investigate the cause of diminished inflammation in Muc-1^KO^ mice, virus titers were analyzed in WT and Muc-1^KO^ spleens at several time points post infection by plaque assay. Viral titers in these strains were indistinguishable on days 2, 12 and 21. Titers in Muc-1^KO^ mice were slightly lower at days 4, 6, and 8, though there was only statistical difference in the titers at day 6 post infection (p.i) (Fig. 1D). In 4 independent experiments, Muc-1^KO^ mice had consistently less inflammatory infiltrate. This trend also held at higher initial doses of virus that achieved higher titers and peak viral titers at days 2–4. This led us to investigate possible roles of Muc-1 in virally-induced pancreatic inflammation.

### CVB3-infected Muc-1^KO^ pancreata have significantly decreased macrophage infiltration

**Figure 2:**
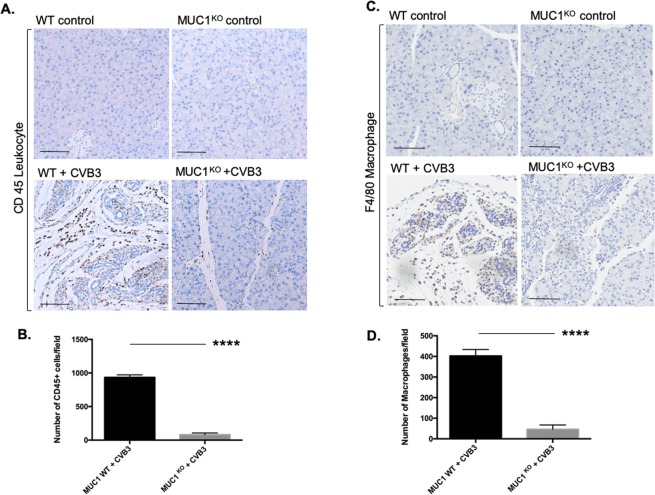
Significant decrease of leukocyte infiltration and macrophage number in Muc-1^KO^ mice in response to CVB3 infection. IHC for (**A**) CD45+ leukocyte and (**C**) F4/80+ macrophages on sections from WT control and Muc-1^KO^ mice with/without CVB3 infection 8-days post-inoculation. Scale bar 50 μm. Quantification of (**B**) CD45+ leukocyte infiltration and (**D**) macrophage recruitment in the pancreas of WT control and Muc-1^KO^ mice with CVB3 infection 8-days post-inoculation. ****p ≤ 0.0001.

Consistent with the histological analysis, there were significantly fewer leukocytes infiltrating the pancreas (IHC staining with CD45, Fig. 2A, Supplementary Fig. 2A,B) in CVB3-infected Muc-1^KO^ mice compared to infected WT mice (Fig. 2B). This suggested that Muc-1^KO^ mice had an impaired inflammatory response to CVB3 infection. As inflammatory monocytes are recruited to most organs, where they give rise to proinflammatory macrophages in response to tissue damage, we evaluated the macrophage population in these animals during the course of infection, production of pancreatitis, and resolution. As expected, large numbers of macrophages were observed in the WT CVB3-infected pancreata from day 2 to day 8 p.i. (Fig. 2C,D, Supplementary Fig. 2C,D). In contrast, very few macrophages were observed in CVB3-infected Muc-1^KO^ pancreata. To investigate the finding that CVB3-infected Muc-1^KO^ mice showed significantly lower infiltration of macrophages into the pancreas, we hypothesized that 1) Muc-1^KO^ mice lacked chemo-attractants for macrophages; or 2) that Muc-1^KO^ macrophages had intrinsic defects in migration.

### CVB3-infected Muc-1^KO^ pancreata produces significantly lower levels of ICAM-1

**Figure 3:**
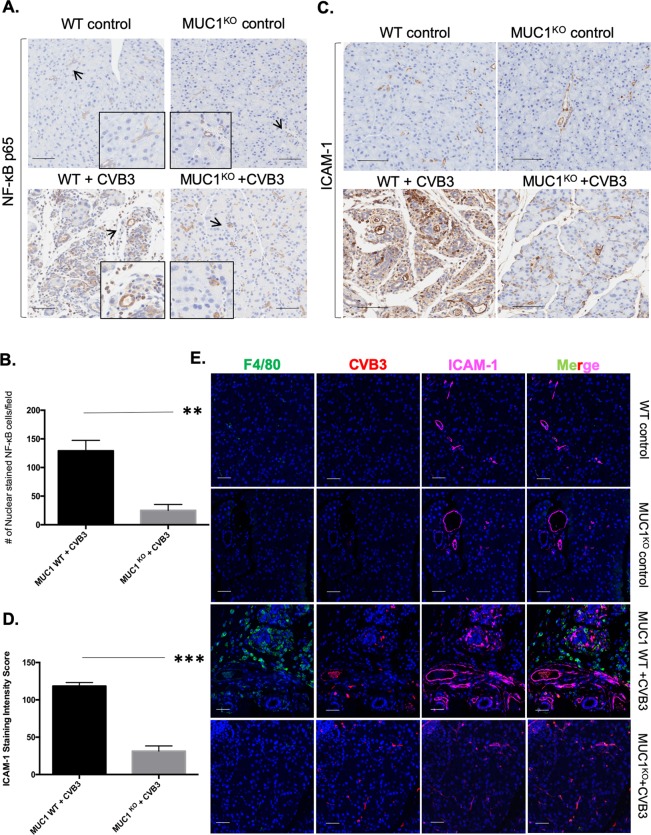
Decreased ICAM-1 expression in pancreas of CVB3-infected Muc-1^KO^ mice compared to pancreas of infected WT mice. IHC staining of (**A**) NFκB p65 and (**C**) ICAM-1 on pancreas sections from WT and Muc-1^KO^ mice with/without CVB3 infection 8-days post-inoculation. Arrows point to the areas represented by the insert. NFκB p65 nuclear staining is observed in infiltrating immune cells and ductal like cells in WT mice that were infected with CVB3. Scale bar 60 μm. Quantification of nuclear-staining (**B**) NFκB p65 positive cells and (**D**) ICAM-1 expression levels collected from CVB3-infected Muc-1 WT and Muc-1^KO^ mice pancreas. **p ≤ 0.01. ***p ≤ 0.001. (**E**) Immunofluorescence co-staining of F4/80 (green), CVB3 (red), and ICAM-1 (magenta) in WT and Muc-1^KO^ mice with/without CVB3 infection 8 days post-inoculation. Blue staining indicates DAPI nuclear staining.

Muc-1 is involved in signaling through proinflammatory pathways that include NF-κB, and these pathways modulate inflammatory conditions in the microenvironment of the pancreas that influence the development of pancreatitis and also the progression of cancer^19–21^. Our data showed that Muc-1^KO^ animals had an approximately 4-fold decrease in the number of cells with nuclear expression of NF-κB, suggesting that knockout of Muc-1 inhibits expression and activation of NF-κB during viral infection (Fig. 3A,B), whereas WT mice exhibit robust NF-κB-mediated pro-inflammatory signaling that results in pancreatitis and consequent histological changes. NF-κB is known to regulate ICAM-1^22,23^, a proinflammatory mediator that recruits macrophages^22,24,25^. Our data show significant elevation (p ≤ 0.001) of ICAM-1 following infection with CVB3 in wildtype animals (Fig. 3C,D). *In vivo*, the distribution of ICAM-1 in relation to CVB3 infected cells and macrophages was informative (Fig. 3E). In pancreas from control mice and Muc-1^KO^ mice, ICAM-1 expression was restricted to blood vessels. However, pancreata from WT mice infected with CVB3 showed abundant expression of ICAM-1 in areas in which the virus was detected, and this area was infiltrated with macrophages in pancreatic tissue from WT mice (Fig. 3E). Taken together, these findings support the hypothesis that Muc-1 influences local NF-κB-mediated proinflammatory signaling, which leads to *de novo* expression of ICAM-1 in the vicinity of viral infection, which in turn enhances macrophage infiltration into the infected and inflamed areas.

### Muc-1^KO^ bone marrow derived macrophages (BMDMs) have impaired migration

**Figure 4:**
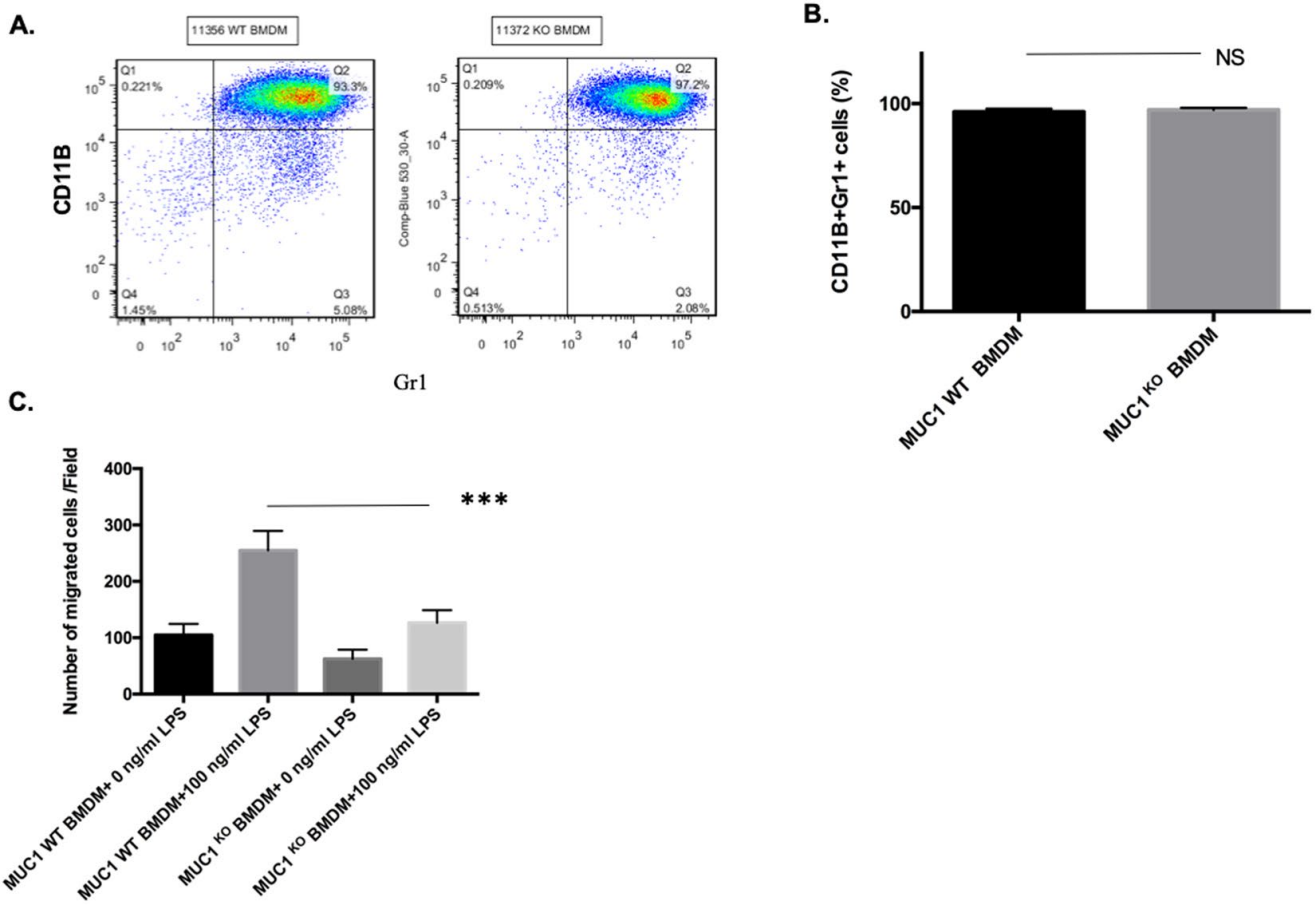
Muc-1^KO^ Bone Marrow Derived Macrophages (BMDM) have impaired migration. (**A**) After 5 days of culture with L929 supernatant medium, BMDM cells from WT and Muc-1^KO^ mice were analyzed for CD11b and Gr1 expression using flow cytometry. (**B**) No significant (NS) difference in monocyte differentiation to macrophages was observed between WT and Muc-1^KO^ mice. (**C**) 1.5 million primary mouse bone marrow derived macrophages were seeded on the upper layer of a transwell chamber and medium with/without LPS was placed below the chamber. After 16-hours incubation period, migrated cells were counted. ***p ≤ 0.001 indicated statistical significance of Muc-1 WT BMDM migration with 100 ng/ml LPS as compared to Muc1^KO^ BMDM migration with 100 ng/ml LPS.

In order to determine if macrophage from the Muc-1^KO^ mice have intrinsic defects in chemotactic migration, *in vitro* migration assays using transwell devices were performed on isolated bone marrow cells cultured with L929 supernatant medium for 5 days in order to promote macrophage differentiation. After differentiation to BMDM, cells from WT and Muc-1^KO^ mice were analyzed for CD11b and Gr1 expression by flow cytometry to determine the uniformity of the cell population with the two genotypes (Fig. 4A). Analysis of the macrophage population showed similar Gr1 and CD11b expression between WT and Muc-1^KO^ mice, which suggests that both WT and Muc-1^KO^ BMDM differentiate normally (Fig. 4B). We then seeded 1.5 million BMDM with culture medium only on the upper layer of a transwell chamber and placed culture medium with/without LPS (100 ng/ml) in the lower chamber to form a chemoattractant gradient. Muc-1^KO^ BMDMs exhibited significantly lower rates of migration than WT BMDM in response to LPS (Fig. 4C). This indicates that Muc-1^KO^ BMDM exhibit defective migration under chemotactic stimulation and this intrinsic defect might partially explain decreased macrophage infiltration during virally-induced pancreatitis in Muc-1^KO^ animals.

### Muc-1^KO^ BMDMs express low levels of CCL2, CCR2 and Integrin VLA-4

**Figure 5:**
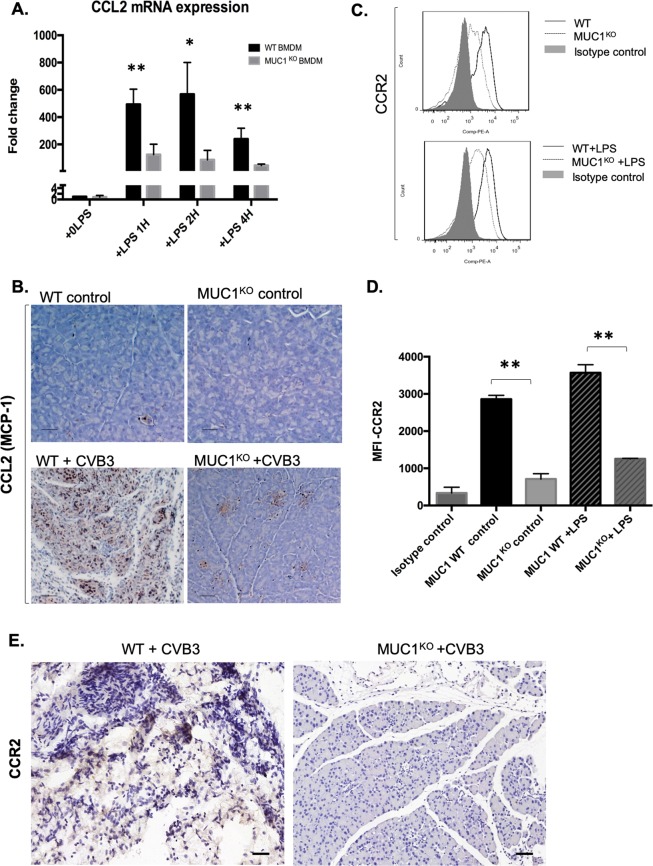
Muc-1^KO^ BMDM express very low levels of the chemokine CCL2 and chemokine receptor CCR2. (**A**) RNA was extracted from WT and Muc-1^KO^ BMDMs stimulated with LPS (100 ng/ml) for 0, 1, 2, or 4 hours. Quantitative RT-PCR was performed and average gene expression as compared to time 0 is shown for CCL2. * p < 0.05 indicates statistical significance of CCL2 mRNA expression level of WT BMDM as compared to the Muc-1^KO^ BMDM at 2 hours and 4 hours LPS treatment time points. (**B**) IHC staining of CCL2 on pancreas sections from WT and Muc-1^KO^ mice with/without CVB3 infection 8-days post-inoculation showed that CCL2 levels were significantly lower in CVB3 infected Muc-1^KO^ mice pancreas compared to WT infected mice. (**C**) BMDM from WT and Muc-1^KO^ mice were treated with or without LPS (100 ng/ml) for 16 hours and cells were processed for flow cytometric analysis. CD11b+Gr1+ cells were gated and the expression level of CCR2 were compared. Flow cytometric data shown are representative of 3 independent experiments. (**D**) Quantitative data showed that the median fluorescent intensity (MFI) of CCR2 expression levels were compared between the WT and Muc-1^KO^ BMDM. Quantification data shown are mean ± SD. **p ≤ 0.01 indicates statistical significance. (**E**) The mRNA expression level of CCR2 were evaluated by real-time PCR in pancreas from WT and Muc-1^KO^ mice with CVB3 infection at 4 days, 6 days and 8 days post-inoculation. **p ≤ 0.01, ****p ≤ 0.0001 indicates statistical significance. (**F**) IHC staining of CCR2 on pancreas sections from WT and Muc-1^KO^ mice with CVB3 infection 8-days post-inoculation.

To further characterize the biology of BMDM from WT and Muc-1^KO^ mice, we assessed the expression of key cytokines in wildtype and Muc-1^KO^ BMDM under conditions of LPS activation. No difference was observed in proinflammatory TNF-α or M-CSF at any time point upon LPS stimulation (Supplementary Fig. 3A). However, the proinflammatory cytokines CCL2, MIP-2 and IL-1β were significantly elevated in WT BMDM as compared to Muc-1^KO^ BMDM (Fig. 5A, Supplementary Fig. 3A). Previous studies have shown that migration of bone marrow (BM) monocytes to inflamed tissue sites is dependent on CCL2/CCR2 signaling^26–29^. We observed that CCL2 was produced at significantly lower levels in Day 8 CVB3-infected Muc-1^KO^ pancreata than in WT pancreata (Fig. 5B). We next examined CCR2 expression in wildtype and Muc-1^KO^ BMDM under conditions of LPS activation. Muc-1^KO^ BMDM also expressed significantly lower levels of CCR2 (Fig. 5C,D). These results were confirmed by real-time PCR experiments that evaluated mRNA expression levels of CCR2 in pancreata from WT and Muc-1^KO^ mice at 4 days, 6 days and 8 days post-inoculation with CVB3 (Fig. 5E). Furthermore, evaluation of CVB3 infected pancreata tissue sections with CCR2 antibody, revealed that CCR2 levels were much lower in CVB3 infected Muc-1^KO^ mice pancreata compared to WT infected mice (Fig. 5F). Taken together, these results suggest that low expression levels of CCL2 and CCR2 may contribute to the impaired migration of Muc-1^KO^ BMDM. As CCR2 expression is induced by Type1 IFN, we hypothesized that type1 IFN and its related chemokines CXCL9, 10, 11 are involved in the development of pancreatitis. Real-time PCR analysis of type1 IFN and chemokines CXCL9, 10, 11 showed that of type1 IFN and CXCL9 are significantly higher in CVB3-infected WT mice pancreata compared to Muc-1^KO^ mice pancreata (Supplementary Fig. 3B). IHC staining of type1 IFN also confirmed that type1 IFN levels were significantly higher in CVB3 infected WT mice compared to Muc-1^KO^ mice pancreata (Supplementary Fig. 3C). These results further support a role of type1 IFN in inducing macrophage infiltration of the pancreas through regulation of the receptor and ligand chemotaxis^30^.

**Figure 6:**
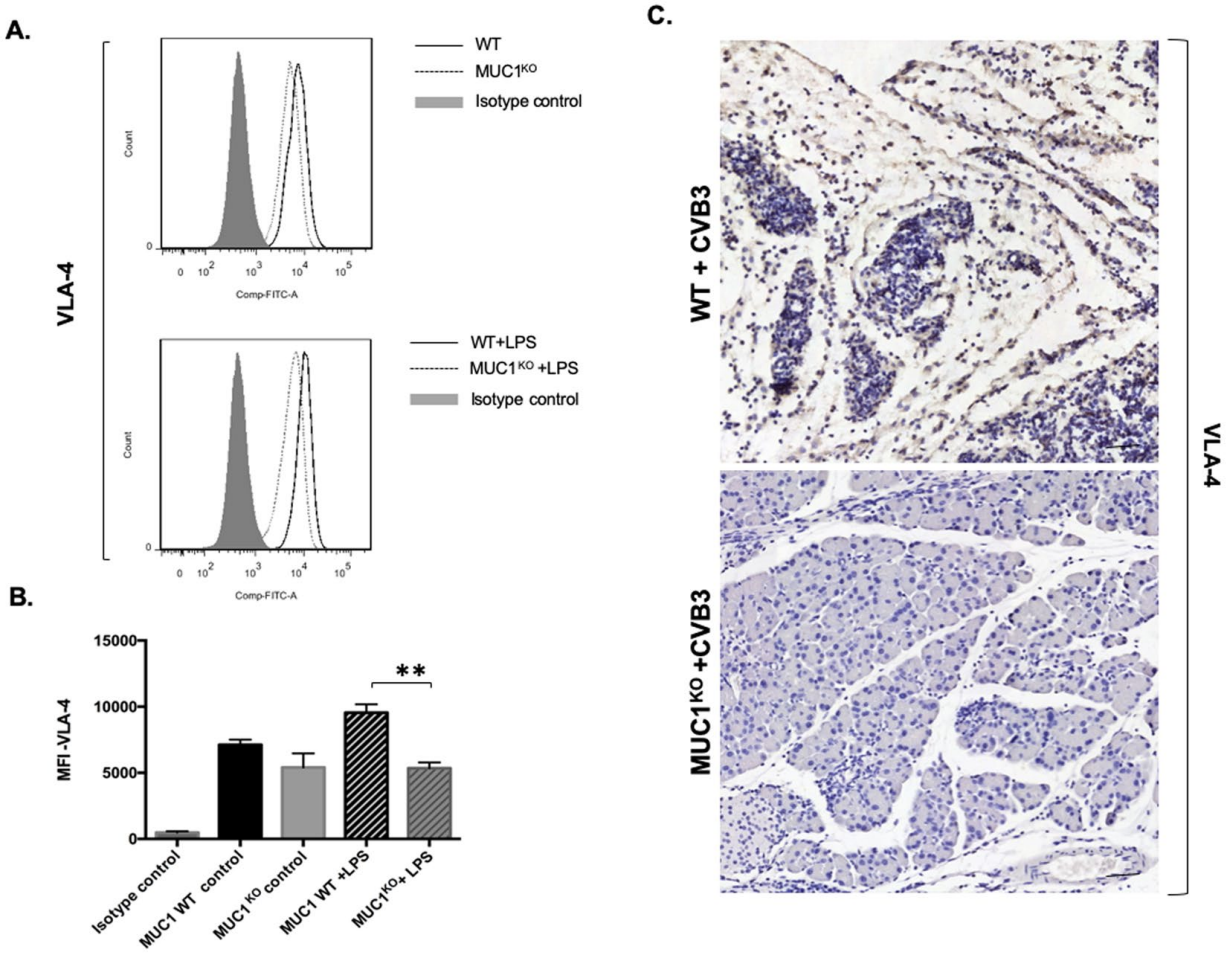
Muc-1^KO^ BMDM express lower levels of the integrin VLA-4. (**A**) BMDMs from WT and Muc-1^KO^ mice were treated with or without LPS (100 ng/ml) for 16 hours and cells were processed for flow cytometric analysis. CD11b+Gr1+ cells were gated and the expression levels of VLA-4 were compared. Flow cytometric data shown are representative of 3 independent experiments. (**B**) Quantitative data showed that the median fluorescent intensity (MFI) of VLA-4 expression levels were compared between the WT and Muc-1^KO^ BMDMs. Quantification data shown are mean ± SD and represent three independent experiments. **p ≤ 0.01 indicates statistical significance. (**C**) IHC staining of VLA-4 on pancreas sections from WT and Muc-1^KO^ mice with CVB3 infection 8-days post-inoculation.

We also examined expression of the adhesion molecule VLA-4, as integrins are known to be important molecules for trans-endothelial migration by macrophages^31,32^. To determine if the expression levels of VLA-4 were different between WT BMDM and Muc-1^KO^ BMDM, we activated WT or Muc-1^KO^ BMDM with LPS and evaluated those cells by flow cytometry for expression levels of VLA-4. Activated WT BMDM had significantly higher expression levels of VLA-4 as compared to Muc-1^KO^ BMDM (Fig. 6A,B). These data indicate that low expression levels of VLA-4 may also contribute to the impaired migration of Muc-1^KO^ BMDM. As a result of impaired migration, we observed very low levels of VLA-4 in CVB3 infected Muc-1^KO^ mice pancreata (Fig. 6C).

## Discussion

We investigated the role of Muc-1 in early stages of a physiologically relevant model of enterovirus-induced pancreatitis. CVB3, a common human pathogen that is associated with acute pancreatitis in humans, induces a similar acute pancreatitis in mice^14,17,33^, which is characterized by the infiltration of inflammatory cells, edema, and acinar cell necrosis. Our results indicate that Muc-1 deficiency significantly attenuated the onset and severity of pancreatitis and decreased the degree of tissue damage that resulted from infection with CVB3. It was notable that the decrease in tissue damage was not due to viral load, as CVB3-infected Muc-1^KO^ mice exhibited viral titers that were sufficient to produce severe pancreatitis in wild-type mice (Fig. 1). Increasing the initial dose of infectious viral particles led to even higher viral titers that did not induce severe pancreatitis in CVB3-infected Muc-1^KO^ mice. CVB3-infected Muc-1^KO^ mice consistently produced slightly lower peak titers of virus over repeated experiments. We attribute this to the fact that inflammation is known to exacerbate viral infectivity and replication, in part by upregulating cellular factors that enhance viral replication^34^. Thus, the decreased pancreatitis we observed in Muc-1^KO^ mice could be due to decreased inflammation in the pancreas during viral infection.

In the course of investigating the nature of the inflammatory response to CVB3, we observed significantly decreased infiltration of immune cells (CD45+), especially macrophages, in CVB3-infected Muc-1^KO^ mice. Macrophage infiltration to the pancreas is an important component of inflammation associated with pancreatitis. Therefore, we investigated two hypotheses to explain decreased macrophage infiltration in this model: that Muc-1^KO^ mice lack key chemo-attractants for macrophage; and that macrophages from Muc-1^KO^ mice have intrinsic defects in migration.

Our initial evaluation of the nature of the inflammatory response in this mouse model implicated NF-κB and ICAM-1 as mediators of virally-induced inflammation in WT animals that was attenuated in Muc-1^KO^ mice. Our data suggest that knockout of Muc-1 inhibits expression and activation of NF-κB during viral infection, whereas wildtype mice exhibit robust NF-κB mediated proinflammatory signaling that results in pancreatitis and consequent inflammatory responses. Activation of NF-κB in epithelial cells of the pancreas is poorly understood. There is evidence that binding of TGFα or epithelial growth factor (EGF) to EGFR can activate NF-κB, but the precise signaling mechanism by which this occurs has never been established. Moreover, EGFR expression has been shown to be required for progression of PanIN lesions to cancer in mice expressing K-ras mutations, under conditions of induction of pancreatitis by caerulein^35^; however, the manner by which EGFR signaling mediates this effect has not been described. There is separate published evidence that EGFR phosphorylates Muc-1 and that Muc-1 transduces signals from the cell surface to the interior of the cells^36,37^, though the full spectrum of targets of this signaling are not defined. Our observation that knockout of Muc-1 attenuates NF-κB expression and activation during CVB3-induced pancreatitis leads us to propose that Muc-1 is a signaling effector between EGFR and NF-κB.

We also observed significantly elevated levels of ICAM-1 and concomitant infiltration of macrophages into areas of the pancreas that showed CVB3 infection in our experiments with WT mice, but these were reduced in Muc-1^KO^ mice. This low level of ICAM-1 expression in CVB3 infected Muc-1^KO^ mice may contribute to low levels of macrophage infiltration in several ways. ICAM-1 has been shown to serve as a chemoattractant for macrophages^24^, and consequently it is possible the lack of *de novo* expression of ICAM-1 in pancreatic cells lacking Muc-1 results in a lack of chemotactic signals upon infection. Secondly, there is published data demonstrating that Muc-1 binds ICAM-1^38,39^ and that Muc-1 expressed on leukocytes mediates transendothelial cellular migration of macrophages by binding to ICAM-1^40,41^, in part by initiating Src-CrlL-Rac1/Cdc42 mediated actin cytoskeletal protrusive motility^42,43^. Interestingly, Src signaling has also been shown to contribute to cerulein-induced murine pancreatitis by stimulating chemokine production and by activating NF-κB in acinar cells^44^. Sustained activation of NF-κB in acinar cells has been shown to result in severe acute pancreatitis with intense local and systemic inflammatory responses^45–47^. Thus, in addition to the chemotaxis mediated by ICAM-1, our results raise the possibility that interactions between Muc-1 and ICAM-1 influence leukocyte motility directly by facilitating transendothelial migration of leukocytes and/or by activating NF-κB signaling in pancreatic cells.

We also tested the hypothesis that Muc-1^KO^ macrophages may have intrinsic defects in chemotactic migration. Fresh BMDMs isolated from WT and Muc-1^KO^ mice were shown by flow cytometry to exhibit appropriate and similar maturation *in vitro*. However, *in vitro* migration assays revealed that Muc-1^KO^ BMDMs had significantly decreased LPS-induced migration as compared to WT BMDM, in part due to the low expression levels of CCR2, CCL2 and VLA-4 by Muc-1^KO^ BMDM. This is consistent with a separate study that showed CCR2 deficient mice treated with a low-dose cerulein regimen to induce pancreatitis exhibited decreased pancreatitis as well as decreased levels of CD11b+ macrophages^48^. High levels of expression of a secreted form of Muc-1 were previously shown to be associated with increased production of CCL2^49^ suggesting Muc-1 can positively regulate CCL2.

In summary, our results demonstrate that elimination of Muc-1 from all cell types in mice significantly reduces inflammation and severity of pancreatitis associated with CVB3 viral infection. Evidence presented here suggests that this results from elimination of the functional role of Muc-1 on multiple cell types including inflammatory leukocytes and cells of the exocrine pancreas. Effects on leukocytes include the possible reduction of cell migratory capability through interactions with ICAM-1, alterations in the expression of chemokines and other adhesion molecules such as VLA-4, and alterations in signaling that affect NF-κB activity. Effects on pancreatic exocrine cells include alterations in cytokine and chemokine production. The relative contribution of these mechanisms to the observed reduction of inflammation and consequent pancreatitis should be further investigated. Our results suggest that molecular inhibition of these functions of Muc-1 offer a heretofore unexplored therapeutic avenue to reducing inflammation associated with pancreatitis.

## Materials and Methods

### Animal models

Muc-1^KO^ mice on a C57BL/6 background were a generous gift from Dr. Sandra Gendler (Mayo Clinic). The mice were generated using homologous recombination as previously described^1^. Age matched Muc-1 wildtype mice were used as controls. All mice were infected by intraperitoneal injection of 5 × 10^5^ tissue infective dose 50 (TCID_50_) of CVB3/CO and the viral titers of infected mice were determined as previously described^10,50^. All mice studies were approved by the University of Nebraska Medical Center IACUC committee. Housing and all animal procedures were performed in accordance with institutional and national regulations.

### Histologic evaluation

Longitudinal sections of pancreas were fixed in 4% paraformaldehyde for 16 hours and paraffin embedded, and 4-μm sections were cut. Sections were stained with H&E. Histologic scores were graded and recorded by two different blinded pathologists. Histologic score was graded on acinar cell loss, edema, and leukocyte infiltration. A score of 1 = 5–25%, 2 = 26–50%, 3 = 51–75%, and 4 = 76–100%.

### Reagents

Lipopolysaccharide (LPS) was purchased from Sigma-Aldrich (St. Louis, MO). Anti-coxsackievirus B3 Antibody (MAB948) was purchased from EMD Millipore (Jaffrey, NH). Anti-F4/80 and anti-NFκB p65 were from Abcam (Cambridge, MA). Anti-ICAM-1 and anti-CCR2-PE antibodies were from R&D Systems (Minneapolis MN). Anti-CD16/32 Fc Block, anti-CD11b-APC/CY7, and anti-VLA-4-FITC were from Biolegend. Anti-Gr1-PE-Cy7 was from BD bioscience (San Jose, CA). Alexa Fluor 488, 594 or 633 labeled secondary antibodies were from Life Technologies (Carlsbad, California). The viability dye Ghost violet 510 was from TonBo. For real-time PCR Power SYBR Green RNA-to-Ct^TM^ 1-Step kit from Thermal Fisher was used.

### Immunohistochemistry and Immunofluorescence

Formalin-fixed tissue specimens were cut into 4-µm sections. These specimens were deparaffinized with xylene and rehydrated with a decreasing gradient of alcohol. Antigen retrieval was performed by boiling the sections in 10 mM citrate buffer (pH 6) for 10 mins. 3% H_2_O_2_ was added to tissue samples for no more than 5 mins, then slides were washed with PBS and blocked with protein block serum-free solution (DAKO, Carpinteria, CA) for 30 mins. Immunohistochemistry was performed with primary antibodies and then secondary antibodies labeled with horseradish peroxidase, which was detected using 3, 3-diaminobenzidine as a chromogen. Slides were counter-stained with hematoxylin and dehydrated with an increasing alcohol gradient ending with xylene. Slides were sealed with mounting media. Images were captured using the panoramic 250 digital slide scanner and Caseviewer software (3DHistech, Hungary). For immunofluorescence, Alexa Fluor 488, 594 or 633 labeled secondary antibodies from Invitrogen (Grand Island, NY) at a 1:500 dilution were added (room temperature, 1 hour). DAPI (nucleus stain, final concentration 125 μg/ml) was added for 15 minutes after samples were incubated with the secondary antibodies. Fluoromount-G (Southern Biotech, Birmingham, AL) was used as mounting and imaging medium. Images were captured by Panoramic 250 digital slide scanner and Caseviewer software (3DHistech, Hungary) with consistent exposure time and settings.

### Bone marrow derived macrophage (BMDM) isolation and migration assay

Bone marrow (BM) was flushed from femurs of C57/BL6 and Muc-1^KO^ mice with BM flushing buffer (1x PBS + 2%FBS). A single-cell suspension of BM cells was created by pipetting. Cells were passed through a 70 µm cell strainer. BM cells were cultured with L-929 conditioned medium for 5–6 days. 1.5 million primary BMDMs were seeded on the upper layer of a transwell chamber insert (5 µm pore; polycarbonate membrane, Corning, NY, USA) and medium with/without LPS (100 ng/ml) was placed below the chamber. Following a 16-hour incubation period, the cells that migrated through the chamber were counted.

### RT-PCR

RNA was extracted from WT and Muc-1^KO^ BMDMs using a standard trizol method. Quantitative Real-time PCR was performed using Power SYBR Green RNA-to-CtTM 1-Step kit according to the manufacturer’s protocol. The fold change in mRNA expression between experimental groups and control groups was analyzed by the standard ^ΔΔ^Ct method. GAPDH was used as an internal control. RT-PCR primer sequences are as follows^51^: M-CSF, TAGAAAGGATTCTATGCTGGG and CTCTTTGTTGAGAGTCTAAG; GM-CSF, CTACTACCAGACATACTGCC and GCATTCAAAGGGGATATCAG; TNFα, CTATGTCTCAGCCTCTTCTC and CATTTGGGAACTTCTCATCC; Ccl5 (Rantes), AGGAGTATTTCTACACCAGC and CAGGGTCAGAATCAAGAAAC; Cxcl2 (MIP-2), GGGTTGACTTCAAGAACATC and CCTTGCCTTTGTTCAGTATC; Ccl2 (MCP-1), CAAGATGATCCCAATGAGTAG and TTGGTGACAAAAACTACAGC; IL-1β, GGATGATGATGATAACCTGC and CATGGAGAATATCACTTGTTGG; IL-6, AAGAAATGATGGATGCTACC and GAGTTTCTGTATCTCTCTGAAG; GAPDH, CCTGGAGAAACCTGCCAAGTATG and AGAGTGGGAGTTGCTGTTGAAGT.

### Flow cytometry

2 million WT or Muc-1^KO^ BMDMs cells were suspended in FACS buffer (1×PBS+ 1%BSA) and incubated with CD16/CD32 Fc Block for 15 minutes at 4 °C. Cells were labeled with viability dye Ghost violet 510 for 15 mins at 4 °C. Cells were washed 2 times with FACS buffer and then APC/CY7-conjugated anti-CD11b, PE/CY7-conjugated anti-Gr-1, PE-conjugated anti-CCR2, and FITC-conjugated anti-VLA-4 antibodies were added for 30 minutes at room temperature. Cells were fixed with 4% PFA for 10 minutes at room temperature. Unstained and single-stained cells and FMO (fluorescence minus one) controls were used as compensation controls. Cells were analyzed on a LSR flow cytometer (BD Biosciences, San Jose, CA), gated for Gr1+/CD11b+ and then analyzed for median fluorescent intensity of CCR2 and VLA-4. Results were analyzed by FlowJo software (FlowJo, Ashland, OR).

### Statistical analysis

Wilcoxon rank sum test was used for comparing the virus titers and severity scores. Data are presented as mean ± SD. The rest of the statistical p values were acquired with the Student’s t test using Prism (GraphPad Software), and p < 0.05 was considered statistically significant.

## Supplementary information

Supplementary information for Mucin-1 is required for Coxsackie Virus B3-induced inflammation in pancreatitis

## Data Availability

All Data generated from current study are available from the corresponding author upon request.
