# Supplementary material for: Mucin-1 is required for Coxsackie Virus B3-induced inflammation in pancreatitis
Source: Sci Rep. 2019 Jul 23;9:10656. doi: 10.1038/s41598-019-46933-y (PMC6650496; doi:10.1038/s41598-019-46933-y)
Supplement: Supplementary file 1 — Supplementary information for Mucin-1 is required for Coxsackie Virus B3-induced inflammation in pancreatitis [file 41598_2019_46933_MOESM1_ESM.pdf]

Supplementary information for

Mucin-1 is required for Coxsackie Virus B3-induced inflammation in pancreatitis

Xiang Liu[1]*, Dahn L. Clemens[2], James A. Grunkemeyer[3], Jeffrey D. Price[1], Kelly O'Connell[4], Nora M. Chapman[5], Peter Storz[6], Haitao Wen[7], Jesse L. Cox[5], Whitney L. Reid[5], Michael A. Hollingsworth[4], and Sarah Thayer[1]

[1] Division of Surgical Oncology, Department of Surgery, Fred and Pamela Buffet Cancer Center, University of Nebraska Medical Center, Omaha, NE, USA;
[2] Department of Internal Medicine, Division of Gastroenterology and Hepatology; University of Nebraska Medical Center, Omaha, NE, USA;
[3] Midwest laboratories, Omaha, NE, University of Nebraska Medical Center, Omaha, NE, USA;
[4] Eppley Institute for Research in Cancer and Allied Diseases, Fred & Pamela Buffett Cancer Center, University of Nebraska Medical Center, Omaha, NE, USA;
[5] Department of Pathology and Microbiology, University of Nebraska Medical Center, Omaha, NE, USA;
[6] Department of Cancer Biology, Mayo Clinic, Jacksonville, FL 32224, USA.
[7] Department of Microbial Infection and Immunity, Infectious Disease Institute, The Ohio State University Comprehensive Cancer Center, The Ohio State University, Columbus, OH 43210, USA

* Correspondence to xli1@unmc.edu

Supplementary Figure 1.

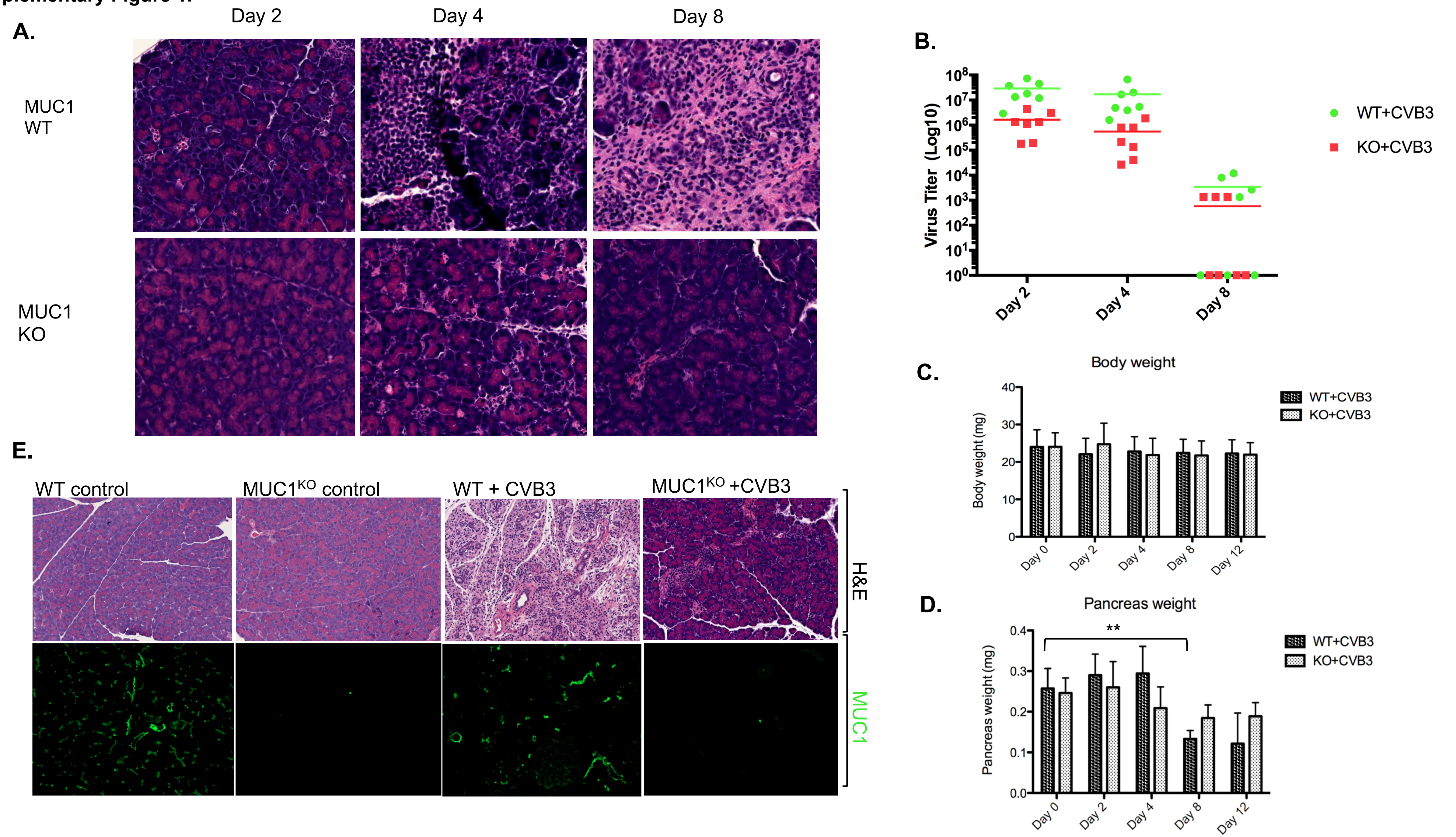

F.

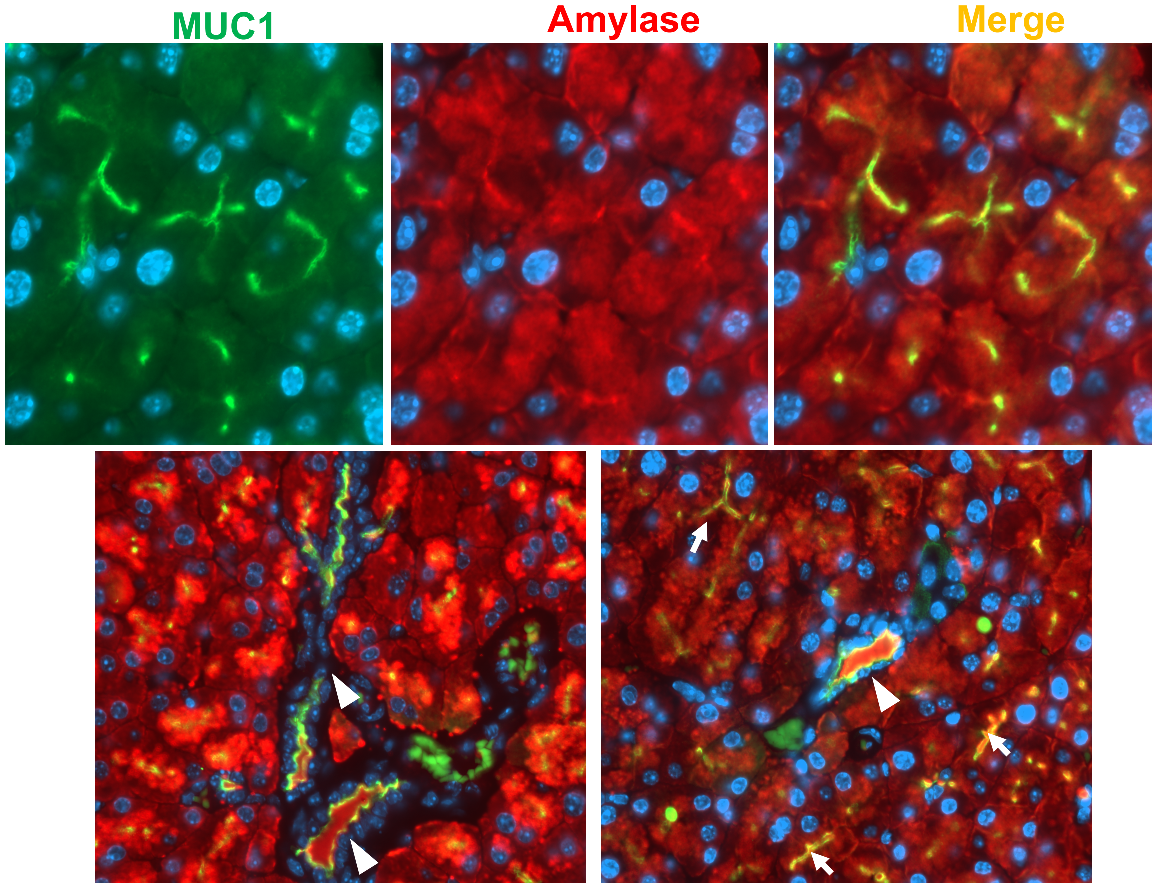

G.

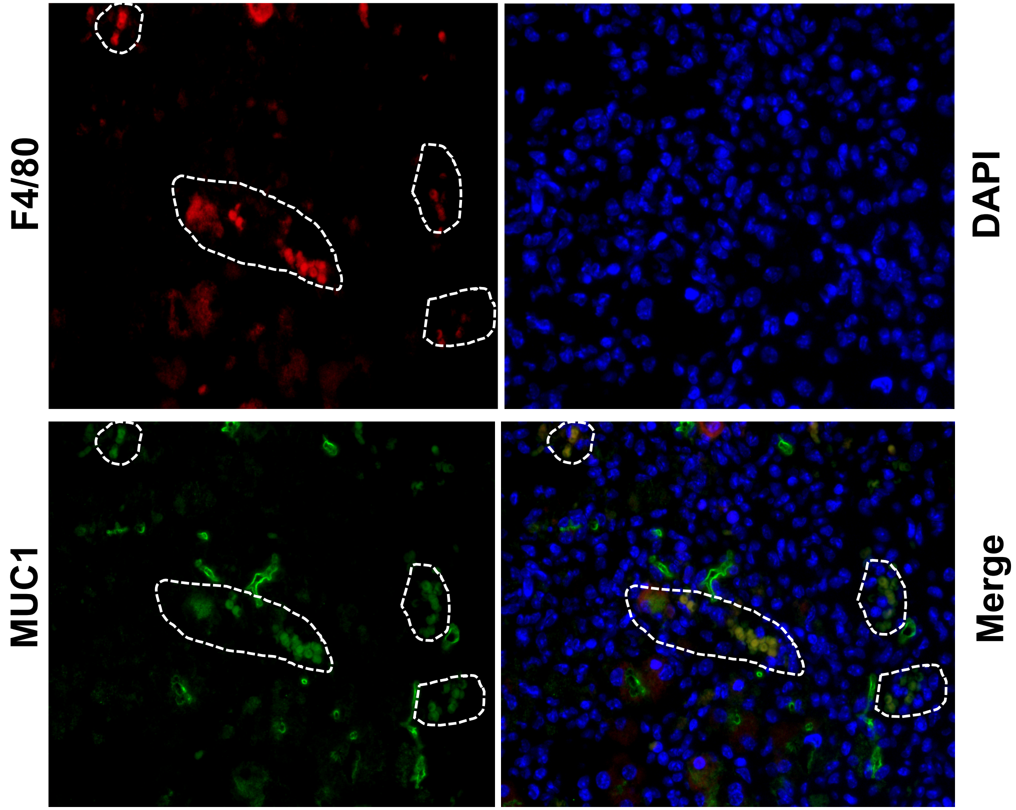

Supplementary Figure 1. Analysis of inflammation, body weight and pancreas weight in CVB3 infected WT and Muc1^{KO} mice. **(A)** Representative H&E-stained sections of pancreas from control mice and Muc1^{KO} mice inoculated with higher dose of virus (10e7 pfu) for 2, 4, 8 days. **(B)** Virus titer from spleens following virus infection at each time point was analyzed by plaque assay. Statistical analysis of Muc1WT and Muc1KO mice body weight **(C)** and pancreas weight **(D)** of WT and Muc1^{KO} mice was assessed during the course of infection at day 0, 2, 4, 8 and 12. ** $p \leq 0.01$ indicates statistical significance. **(E)** Pancreas from WT control, Muc1^{KO} with no CVB3, WT+CVB3 and Muc1^{KO}+CVB3 were subjected to H&E staining and immunofluorescence staining for Muc1. **(F)** Immunofluorescence pictures of MUC1 staining in wildtype mice pancreas. Green indicates MUC1 staining. Red indicates amylase staining. Arrow shows intercalated duct. Arrow heads points to intralobular duct. **(G)** MUC1 expression in macrophages. Red indicates F4/80 staining for macrophages. Green indicates MUC1 staining.

Supplementary Figure 2.

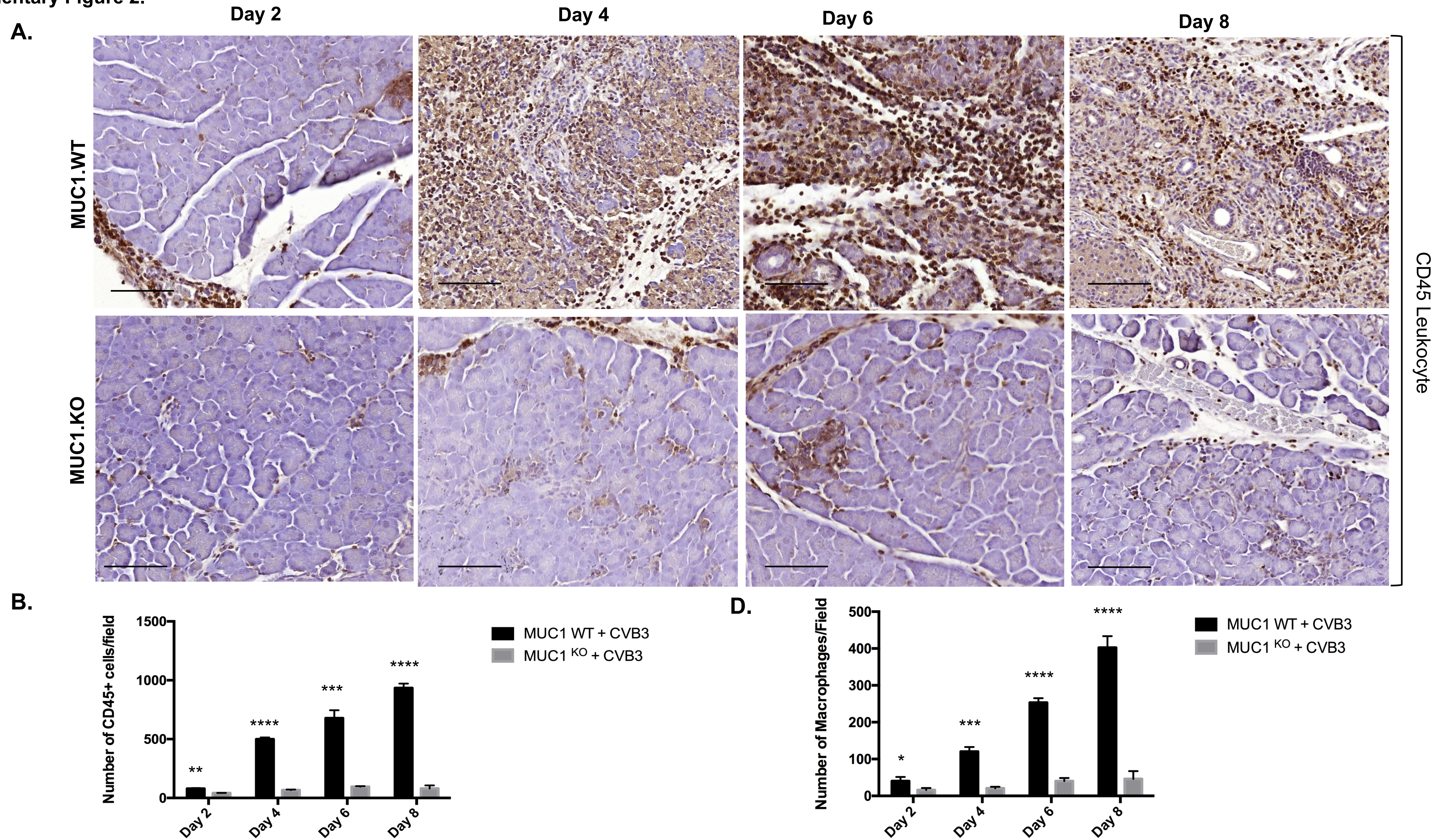

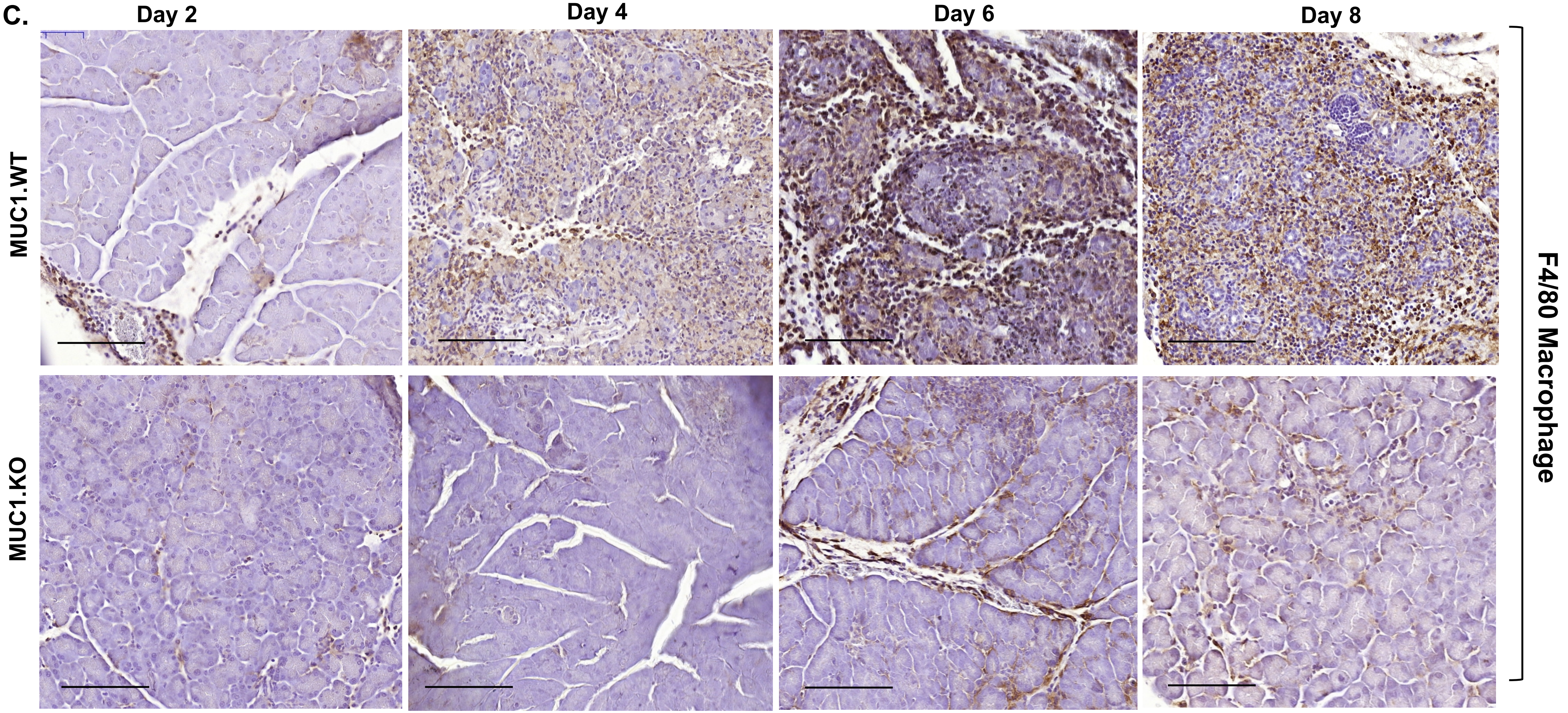

Supplementary Figure 2. Analysis of leukocyte infiltration in CVB3 infected WT and Muc1^{KO} mice. IHC staining of **(A)** CD45+ leukocytes, and **(C)** F4/80+ macrophages in CVB3-infected WT and Muc1^{KO} mice during the course of infection (day 2, 4, 6, and 8). Quantification of **(B)** CD45+ leukocyte infiltration, and **(D)** macrophage recruitment in the pancreas of WT and Muc1^{KO} mice with CVB3 infection on day 2, day 4, day 6 and day 8. * $p < 0.05$, *** $p \leq 0.001$, **** $p \leq 0.0001$ indicates statistical significance.

Supplementary Figure 3.

A.

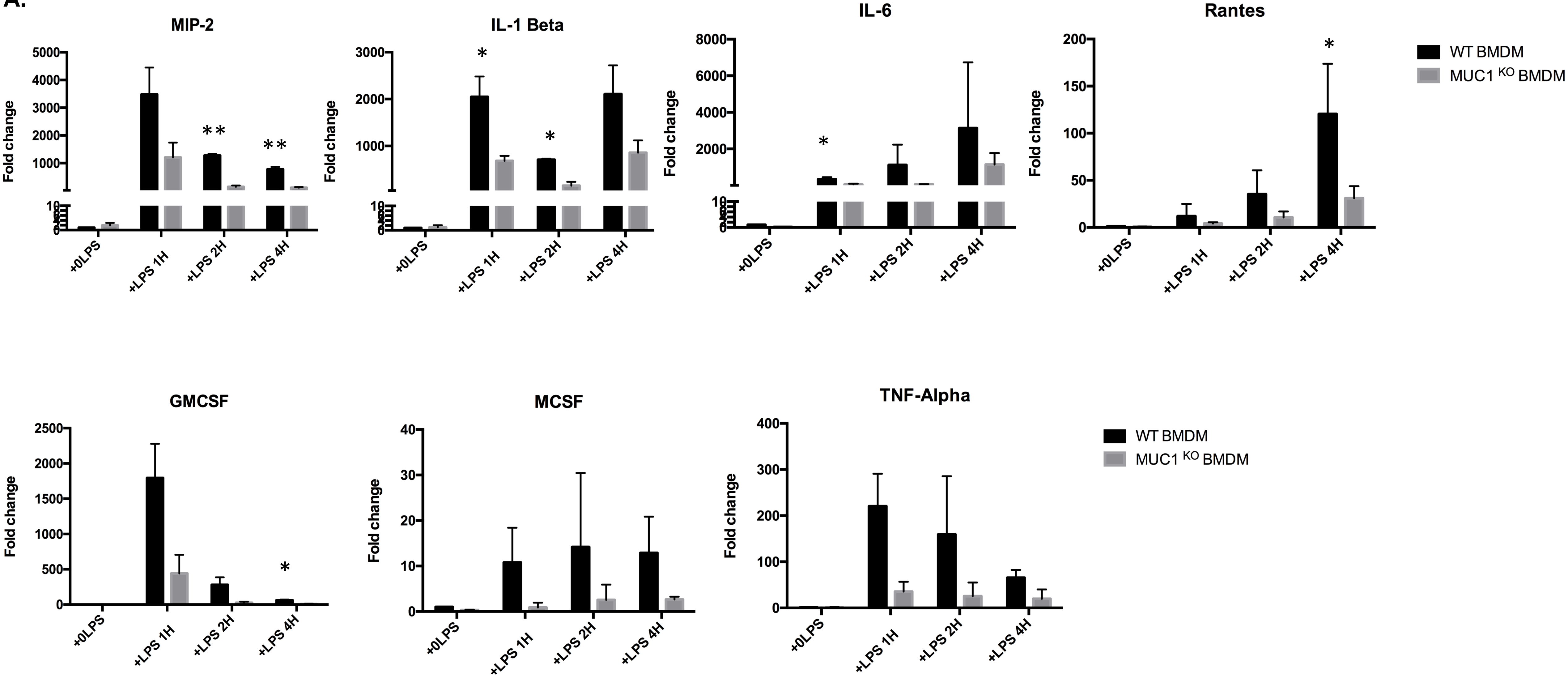

B.

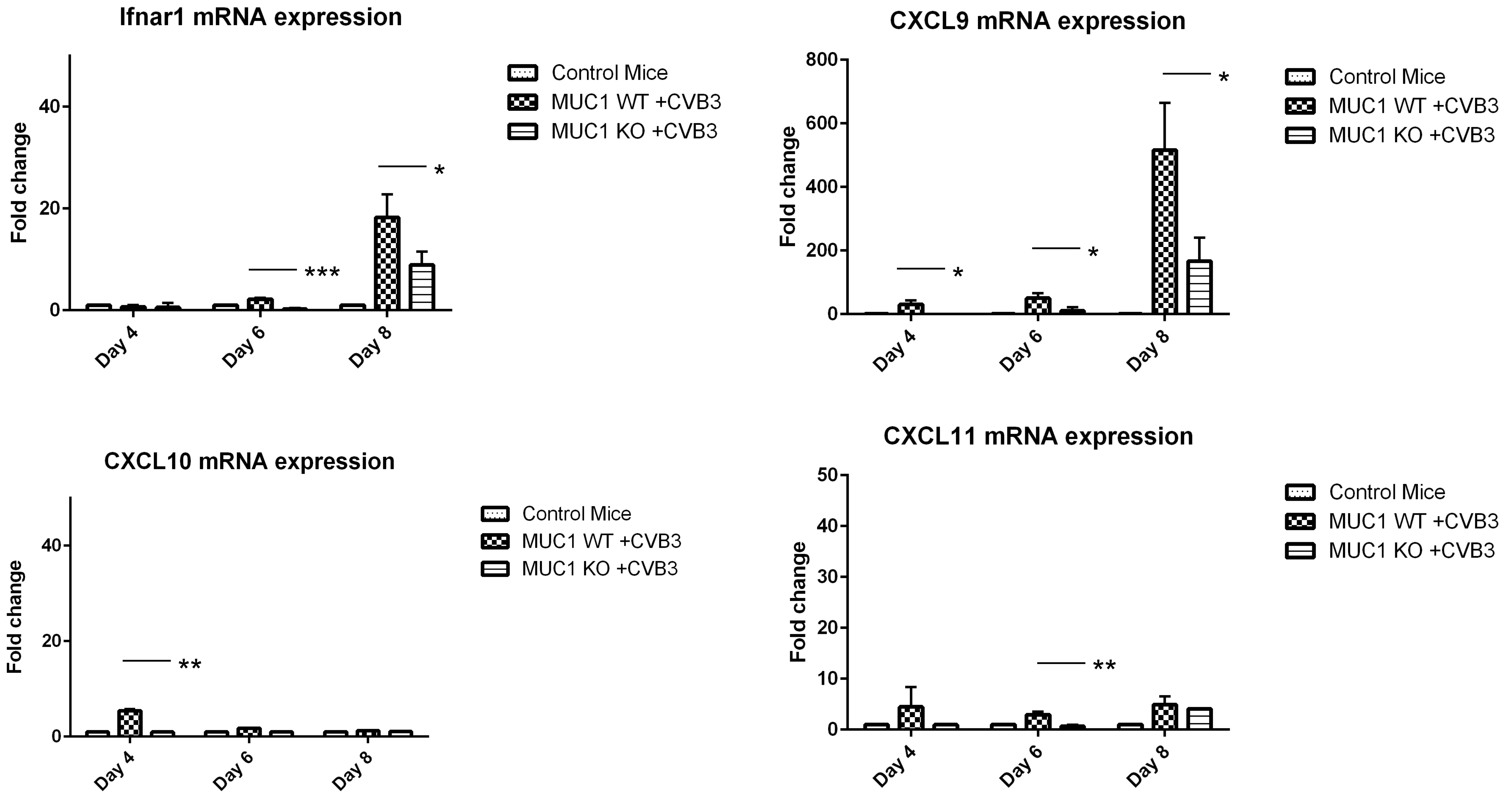
Ifnar1 mRNA expression
Fold change
0
20
40
Day 4
Day 6
Day 8
***
*
Control Mice
MUC1 WT +CVB3
MUC1 KO +CVB3
CXCL9 mRNA expression
Fold change
0
200
400
600
800
Day 4
Day 6
Day 8
*
*
*
Control Mice
MUC1 WT +CVB3
MUC1 KO +CVB3
CXCL10 mRNA expression
Fold change
0
20
40
Day 4
Day 6
Day 8
**
Control Mice
MUC1 WT +CVB3
MUC1 KO +CVB3
CXCL11 mRNA expression
Fold change
0
10
20
30
40
50
Day 4
Day 6
Day 8
**
Control Mice
MUC1 WT +CVB3
MUC1 KO +CVB3

C.

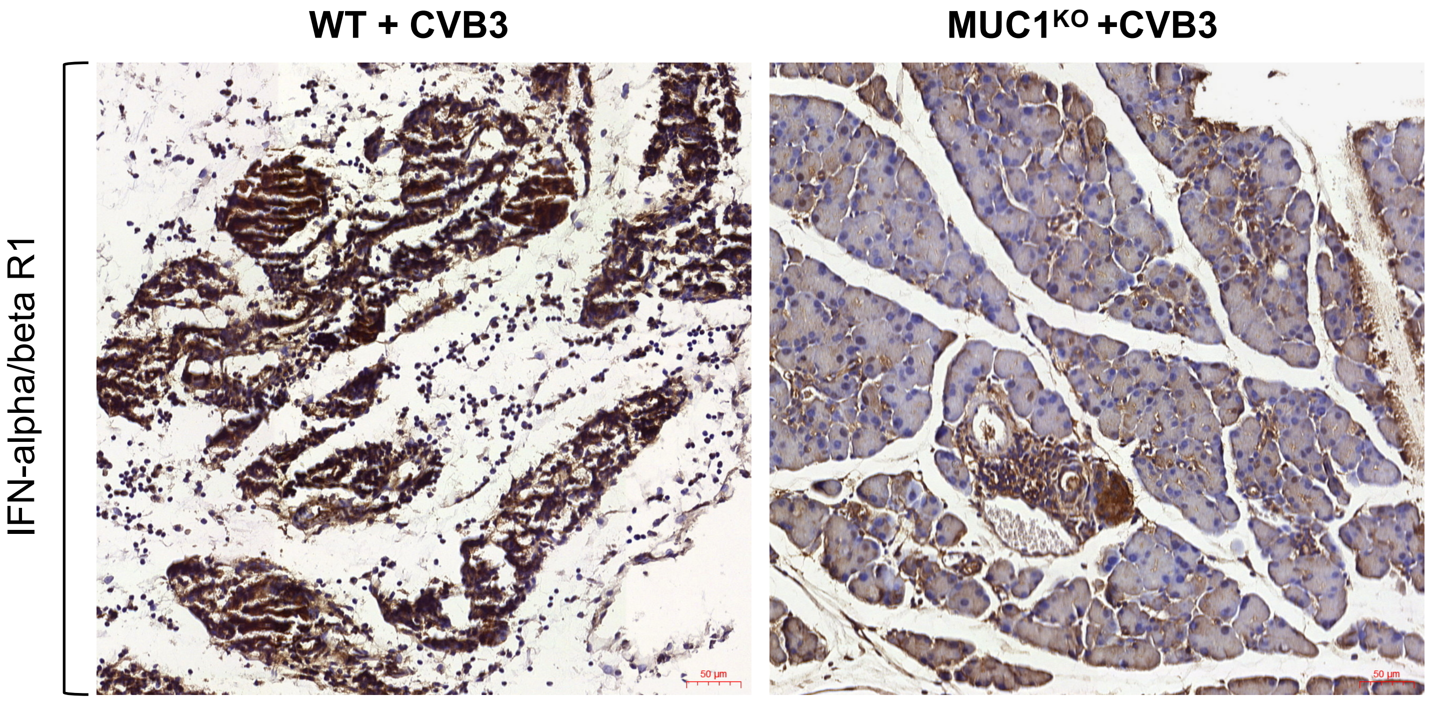

Supplementary Figure 3. Cytokines expression evaluation. **(A)** Cytokines in WT and Muc1^{KO} BMDM were quantified by real-time PCR at different time point upon LPS stimulation. MIP-2: Macrophage Inflammatory protein. Quantification data shown are mean ± SD and represent three independent experiments. ** $p \leq 0.01$ indicates statistical significance. **(B)** The mRNA expression level of type1 IFNs and type1 IFN-related chemokines (CXCL9, 10, 11) were evaluated by real-time PCR in this virus model system. $p < 0.05$, ** $p \leq 0.01$, *** $p \leq 0.001$ indicates statistical significance. **(C)** IHC for type1 IFNs on sections from WT control and Muc1^{KO} mice with CVB3 infection 8-days post-Inoculation. Scale bar 50 μm.
